# High-Performance Room-Temperature NO_2_ Gas Sensor Based on Au-Loaded SnO_2_ Nanowires under UV Light Activation

**DOI:** 10.3390/nano12224062

**Published:** 2022-11-18

**Authors:** Bo Zhang, Shuai Zhang, Yi Xia, Pingping Yu, Yin Xu, Yue Dong, Qufu Wei, Jing Wang

**Affiliations:** 1 Engineering Research Center of IoT Technology Applications (Ministry of Education), Department of Electronic Engineering, Institute of Advanced Technology, Jiangnan University, 1800 Lihu Avenue, Wuxi 214122, China; 2 Research Center for Analysis and Measurement, Analytic & Testing Research Center of Yunnan, Kunming University of Science and Technology, Kunming 650093, China; 3 Key Laboratory of Eco-Textiles (Ministry of Education), Jiangnan University, 1800 Lihu Avenue, Wuxi 214122, China; 4 Key Laboratory of Synthetic and Biological Colloids (Ministry of Education), School of Chemical and Material Engineering, Jiangnan University, 1800 Lihu Avenue, Wuxi 214122, China

**Keywords:** Au-loaded, UV irradiation, synergistic effect, NO_2_, gas sensor

## Abstract

Optical excitation is widely acknowledged as one of the most effective means of balancing sensor responses and response/recovery properties at room temperature (RT, 25 °C). Moreover, noble metals have been proven to be suitable as photosensitizers for optical excitation. Localized surface plasmon resonance (LSPR) determines the liberalization of quasi-free electrons in noble metals under light irradiation, and numerous injected electrons in semiconductors will greatly promote the generation of chemisorbed oxygen, thus elevating the sensor response. In this study, pure SnO_2_ and Au/SnO_2_ nanowires (NWs) were successfully synthesized through the electrospinning method and validated using XRD, EDS, HRTEM, and XPS. Although a Schottky barrier led to a much higher initial resistance of the Au/SnO_2_ composite compared with pure SnO_2_ at RT in the dark, the photoinduced resistance of the Au/SnO_2_ composite became lower than that of pure SnO_2_ under UV irradiation with the same intensity, which confirmed the effect of LSPR. Furthermore, when used as sensing materials, a detailed comparison between the sensing properties of pure SnO_2_ and Au/SnO_2_ composite toward NO_2_ in the dark and under UV irradiation highlighted the crucial role of the LSPR effects. In particular, the response of Au/SnO_2_ NWs toward 5 ppm NO_2_ could reach 65 at RT under UV irradiation, and the response/recovery time was only 82/42 s, which far exceeded those under Au modification-only or optical excitation-only. Finally, the gas-sensing mechanism corresponding to the change in sensor performance in each case was systematically proposed.

## 1. Introduction

NO_2_, one of the most typical and active oxidizing gases, has been thoroughly studied as a target gas in terms of gas sensing. On the one hand, due to the wide presence, large reserves, and great environmental harm caused by NO_2_ [1], relevant research on sensing is of great significance. On the other hand, the high activity and strong oxidizability make NO_2_ more prone to gas-sensitive reactions, further stimulating the interests of researchers [2]. 

As is the case of other target gases, with the broadening of NO_2_ sensing research, the attention on gas sensitivity indicators has changed from a high response [3] to a low operating temperature [4,5,6,7], to equal emphasis on these two indicators [8,9,10]. Nowadays, with the increasing pursuit of low-temperature detection, the resulting low response and lengthy response/recovery times are worrying and need prompt solutions. For NO_2_, its superior electron-withdrawing ability makes it adsorb on the surface of sensitive materials in the forms of NO_2_^−^ and NO_3_^−^ through the direct extraction of electrons from conduction bands [11,12,13,14], which is markedly different from other gases. Obviously, the above mechanisms determine the difficulties in desorption for NO_2_ without thermal excitation compared with other gases [15]. 

In order to achieve better NO_2_ sensing, diverse sensitive material types and supplementary means have been exploited in recent years. Combing the pertinent literature, the main methods can be roughly generalized into three categories when optimizing the sensing performance of a single semiconductor metal oxide (SMO) on NO_2_, namely, (a) noble metal loading [16,17,18,19,20,21,22,23], (b) composites with carbonaceous materials [11,12,13,14,24,25,26,27,28,29,30,31], and (c) optical excitation [32,33,34,35,36,37,38,39,40,41].

As for noble metal (Ag [16], Au [17,18,19,20,21,22,23], Pd [19,20], and Pt [20], etc.) loading, the intrinsic catalytic action of noble metals plays a critical role in improving the NO_2_ sensing properties of corresponding metal oxides. Three universal rules can be determined in the noble metal loading method: (i) the lifting ranges of sensor responses toward NO_2_ for bare SMOs are bound up with noble metal species [17,19,20] and contents [17,21]; (ii) noble metal loading contributes finitely to decreases in sensor operating temperature, and sensors based on noble metal/SMOs composites usually still work at high temperatures when detecting NO_2_ [16,17,18,19,20,21,22,23]; (iii) the catalytic effects of noble metals play a limited role in the improvement of response/recovery speeds for NO_2_ sensing [16,17,18,22,23]. 

Regarding composites with carbonaceous materials (graphene [11,12,13,14,24,25,26,27,28,29,30], CNTs [31], etc.), their inherent good conductivity greatly improves the electrical (conduction) characteristics of SMOs. The conductance modulation of SMOs through carbonaceous materials usually leads to the following consequences concerning NO_2_ sensing properties: (i) carbonaceous materials generally yield composites that exhibit room-temperature NO_2_ gas sensing [11,12,13,14,24,25,26,27,28,29,30,31], and RT is usually the optimal working temperature as well [11,12,29]; (ii) the responses of sensors based on composites consisting of carbonaceous materials and SMOs toward NO_2_ are fairly low at RT [12,13,14,26,27,28,29,30,31]; and (iii) response and recovery processes, especially recovery, in NO_2_ sensing are too lengthy at RT even under the promotion of the high carrier mobility of carbonaceous materials [11,12,24,25,26,28,31]. 

As for optical excitation, distinguished from the two above methods, photoinduced separation of electron–hole pairs will generate numerous free electrons, which eventually increase the content of chemisorbed oxygen species to boost sensing-associated redox reactions [38,42,43]. To some extent, the effect of photon energy injection through photoexcitation can be analogous to traditional thermal activation in determining the occurrence of gas-sensitive reactions [32,34,37]. Most importantly, optical irradiation only causes a slight increase in device temperature [32], which is completely negligible. To date, photoexcitation is regarded as the most effective way to assist the implementation of room-temperature NO_2_ sensing, displacing the original role of thermal activation in this process without increasing the device temperature. In addition, photoexcitation light sources can be further subdivided into visible light [32,33,34,35] or UV irradiation [36,37,38,39,40,41] based on the size (narrow or wide) of the SMO bandgaps. 

Either way, five important conclusions can be summarized: (i) photoactivation plays overlapping and conflicting roles with thermal activation in NO_2_ sensing. Thus, extra increases or decreases in device temperature will suppress the existing optimal responses [32,33,36]. Additionally, the optimal working temperature of light-enhanced NO_2_ sensors (usually RT [33,34,35,36,37,38,39,40,41]) is an eclectic result of corresponding optical irradiation parameters. (ii) The irradiance (intensity) of light sources influences the surface reaction kinetics [37]. Thereby, analogously to familiar optimal operating temperatures, there are also optimal light intensities [32,33,36,37,39] in determining the maximum sensor responses when detecting NO_2_ at RT. Moreover, the optimal irradiance of light-activated NO_2_ sensors is moderate, usually not exceeding 10 mW/cm^2^ [33,36,37,38,39]. (iii) The irradiance of optical irradiation has no intrinsic or routine connection with response time in NO_2_ sensing [44], whereas high-intensity optical irradiation is equal to high-temperature thermal activation and can greatly shorten the recovery time [44,45] of light-enhanced NO_2_ sensors. (iv) Theoretically, wavelength-resonant excitation is most conducive to the maximization of light-activated NO_2_ sensing responses [32,34,35,37], i.e., optical irradiation with photon energy just above the bandgap of sensing material is most favorable to sensor responses. In particular, photons with excessive energies will intensify the inelastic scattering of electrons, thus reducing the charge mobilization efficiency [37]. (v) Overall, photoexcitation can yield a real improvement in NO_2_ sensing, including sensor responses in the noble metal loading method, sensor working temperatures in methods involving composites with carbonaceous materials, and response/recovery speeds, which are significantly superior to both of these methods.

Through the above specific analysis and comparison, optical excitation is optimal among three independent methods in terms of optimizing the NO_2_ sensing performance of SMOs. In fact, paired combinations of any two of the above three methods have also aroused the interest of researchers, considering the possible breakthroughs brought by mutually synergistic effects. Conceivably, there are three modes of combination: (d) consisting of (a) + (b); (e) consisting of (b) + (c); and (f) consisting of (a) + (c).

As for method (d) [46,47,48,49,50,51], carbonaceous materials usually play a decisive role in these hybrid systems. Thus, the collaborative optimization of noble metals and carbonaceous materials on the NO_2_ sensing performance of SMOs embodies more features of method (b) [46,47,48,49]. At RT, the catalytic effects of noble metals are greatly suppressed without the support of thermal activation, being unable to effectively improve sensor responses and response/recovery speeds in NO_2_ sensing [46,47,49]. The synergy of this kind, which inhibits noble metal effects, is not thought to be ideal. 

As for method (e) [52,53,54,55], on the premise of the superior optimization effect on NO_2_ sensing performance brought by optical excitation compared with that by carbonaceous materials, the contribution proportion of carbonaceous materials in this system can hardly be identified when a comparable NO_2_ sensing performance to that only with the assistance of optical excitation is achieved [52,53,55]. Furthermore, in some studies [54], photoexcitation with high irradiance, acting as an accelerant, has been applied only in the recovery process to ameliorate its long duration, which is a feature of room-temperature NO_2_ sensors based on carbonaceous material/SMO composites. The above case further affirms the weak interactions between carbonaceous materials and optical excitation. Hence, the alleged synergistic effects in method (e) are vague and the effectiveness and necessity of this approach are not recognized either. 

Metal nanoparticles, especially noble metals, can strongly absorb light with wide ranges at ambient temperatures [56,57], which significantly strengthens their intrinsic catalytic effects and accelerates the process of corresponding reactions [57]. For plasmonic–metals (Au, Ag, Cu, etc.) [56,57,58,59], resonant collective oscillations of the quasi-free electrons will occur when the frequency of irradiated light matches their natural oscillating frequency [60,61], namely, the well-known LSPR effect [56,57,58,59,60,61]. This mechanism will result in a strong optical resonance extinction (i.e., absorption and scattering) and the generation of abundant hot (activated) electrons [57,61]. For non-plasmonic metals (Pd, Pt, Rh, etc.) [56,57,58,59], light absorption by these metals is mainly through bound electrons [56,57], exciting individual electrons to higher energy levels via interband transitions [56,57,61]. Similarly, strong optical extinction and free electron formation will occur. Therefore, the subsistent synergistic effects between optical excitation and noble metal loading are affirmed. In theory, the additional numerous free electrons make this mechanism extremely conceivable in sensing applications [58,59,60]. 

Supported by this theory, method (f) possesses all reasons and conditions to become the most valuable candidate among the listed methods. In fact, researchers, including our group [56,62], have carried out relevant research on the room-temperature NO_2_ sensing of SMOs functionalized with plasmonic Au [62,63,64,65,66,67], Ag [67,68,69], non-plasmonic Pd [44,56,66], and Pt [44], achieving some interesting results. Among them, Au exhibits the prominent LSPR effect. To the best of our knowledge, until now, there has been limited research on photoexcited SnO_2_-based RT NO_2_ sensors. Herein, plasmonic Au was utilized to form a composite with wide-bandgap SnO_2_. Next, RT sensing properties of pure SnO_2_ and Au/SnO_2_ composite toward NO_2_ in the dark and under UV irradiation were compared and discussed in detail. In this process, the LSPR effect between Au and UV irradiation was verified, which greatly enhanced the sensor response and improved the response/recovery properties.

## 2. Experimental Section

### 2.1. Materials

All the experimental materials, including tin tetrachloride pentahydrate (SnCl_4_·5H_2_O, Aladdin Inc., Shanghai, China), chloroauric acid tetrahydrate (HAuCl_4_·4H_2_O, Aladdin Inc., Shanghai, China), N,N-dimethylformamide (DMF, Aladdin Inc., Shanghai, China), and polyvinylpyrrolidone (PVP, Mw = 1,300,000, Aladdin Inc., Shanghai, China), were of analytical grade or above and used as received without further purification.

### 2.2. Synthesis of SnO_2_ and Au/SnO_2_ NWs

The preparation of SnO_2_ and Au/SnO_2_ NWs was by means of the electrospinning method. In view of the similarity of their synthesis processes, the synthetic procedure of Au/SnO_2_ NWs is presented as an example and described as follows. 

In a typical experiment, 0.35 g (1 mmol) of SnCl_4_·5H_2_O was dissolved into 5 mL of DMF. After continued stirring for 30 min, 100 μL of HAuCl_4_·4H_2_O (20 mg/mL) and 0.6 g of PVP were successively added to the above solution. After sealing and shading treatments, the mixed solution was stirred overnight to form a homogeneous and viscous precursor solution prepared for electrospinning. The precursor solution was transferred to a 10 mL disposable syringe. After the installation of a specialized needle for electrospinning, the syringe was fastened to the propeller of the electrospinning instrument. The high-potential output and ground terminals of the high-voltage DC power supply were connected to the needle and reserved protrusion of the metal roller, respectively. For this system, relevant experimental parameters were approximated first and finally determined: voltage of DC power supply, 11 kV; the advance speed of the propeller, 0.3 mL/h; distance between the spinneret and roller collector, 15 cm; and ambient humidity, 35% RH. After 6 h of electrospinning, which ensured sufficient samples, several layers of nearly white film were wrapped around the tinfoil precoated on the roller. The obtained sample was scrupulously stripped off with tweezers to maintain the film intactness and subsequently transferred to a clean cuboid porcelain boat with a cover. Flake-shaped Au/SnO_2_ NWs were finally obtained after a calcination process in a muffle furnace of the sample encapsulated in the porcelain boat. The calcination parameters were set as follows: the heating rate, 2 °C/min; sintering temperature, 600 °C; and holding time, 2 h. It is necessary to point out that the molar ratio of Au to Sn in the composite was estimated to be 0.5%.

Analogically, when HAuCl_4_·4H_2_O was absent in the raw material, SnO_2_ NWs could be accurately synthesized. In addition, on account of the minor alterations to reaction systems, experimental parameters related to SnO_2_ and Au/SnO_2_ NWs were almost identical. 

### 2.3. Characterization

X-ray powder diffraction (XRD) analysis was carried out on a D/max-2550 X-ray diffractometer (Rigaku Inc., Akishima-shi, Japan) with high-intensity CuKα (λ = 0.154 nm) radiation in the range of 5–90° (2θ). Field emission scanning electron microscopy (FESEM) and energy-dispersive X-ray spectrometry (EDS) images were acquired on a Gemini 500 microscope (ZEISS Inc., Oberkochen, Germany) operating at 15–20 kV. Transmission electron microscopy (TEM) and high-resolution TEM (HRTEM) images were obtained on a JEM-2100F microscope (JEOL Inc., Akishima-shi, Japan) with an accelerating voltage of 200 kV. The X-ray photoelectron spectroscopy (XPS) data were recorded on a K-Alpha system (Thermo Scientific Inc., Waltham, MA, USA). 

### 2.4. Fabrication and Measurement of Gas Sensors

In this study, a classic tubular ceramic gas sensor was adopted, following the established manufacturing procedures briefly described as follows [45]. 

First, a small synthetic powder sample was taken to a mortar and moderate deionized water was added. The mixture was fully but gently ground until it turned into a paste. Then, a little paste was dipped and evenly coated on an alumina ceramic tube (4 mm in length, 1.2 mm in external diameter, and 0.8 mm in internal diameter) with an ink brush. The ceramic tube covered with a thin layer of hydrous paste was placed under an infrared lamp to remove moisture. This process was repeated several times to eventually form a dense and uniform film. Next, the manufactured tubular component was placed in an oven and annealed at 150 °C for 3 h to fully remove the residual water in the film. An alloy coil was passed through the ceramic tube to act as a heat source for the device, and the heating temperature was controlled by adjusting the current of the external DC power supply. Finally, two pins of the heating coil and four pins of the ceramic tube were welded at the sensor socket. Conventionally, several gas sensors were fabricated in parallel with the same material to ensure the objectivity and accuracy of gas-sensing test results.

The gas-sensing properties of SnO_2_ and Au/SnO_2_ NWs were evaluated through a self-built gas-sensing test system under laboratory conditions (30% RH, RT). The construction method of the test system and subsequent testing process are summarized as follows. 

First, at the gas supply end, two gas source categories needed to be prepared (drying air and target gas: NO_2_, for example) and stored in their own cylinders. One tee-junction, several mass-flow gas meters, sufficient pneumatic pipes, and specialized connectors were also needed. Drying air mainly acted as the carrier gas to dilute target gas to a certain concentration. Drying air and target gas were directed through independent gas piping, in which a gas meter was embedded, to two joints of the tee-junction. After a transitory confluence, the gas mixture flowed forward to a customized cylindroid quartz bottle, with one side tube at its top and bottom. In a typical experiment, test gases with desired concentrations could ultimately be obtained in the quartz bottle through the flexible matching of two gas flow rates. 

At the signal acquisition end, a sensor base, a perforated rubber stopper, DuPont threads, commercial LED point lamps, and other necessary accessories needed to be prepared. One end of DuPont threads, passing through the rubber stopper, was welded to the bases of the sensor component and the LED lamp. Here, the base of the LED lamp faced upwards and was about 3 cm directly below the sensor base, ensuring direct irradiation of the LED lamp on the sensing film. In addition, all of the above components were supported and fixed using metal wires and melt adhesive to maintain the steadiness of the system. The other ends of the DuPont threads were correspondingly connected to a 8846A desktop multimeter (Fluke Inc., Everett, WA, USA) and two GPD-4303S DC power supplies (GW Instek Inc., Xinbei, China), based on the specific function of each set of test lines. After the insertion of as-prepared gas sensors in the sensor base, electrical signals of sensing materials under different conditions (target gas, temperature, or light irradiation) were recorded by the multimeter and displayed in real-time through the testing software installed on a computer.

Specifically, LED point lamps (Xusheng Inc., Shenzhen, China) used in this study were purchased. these were mass-produced and of low cost. The selected series of LED lamps with quartz encapsulation emitted ultraviolet light, whose wavelength ranges were identical (365–370 nm) but the lamp powers were discrete (0.5, 1, 1.5, 3, and 5 W). Light intensities (irradiances) corresponding to each lamp power were confirmed through a PM16-120 digital optical power meter (THORLABS Inc., Newton, NJ, USA), keeping the distance between the lamp and power meter about 3 cm, the same as above, to be roughly 0.15, 0.27, 0.42, 0.93, and 1.46 mW/cm^2^. When LED lamps with different powers were freely switched, the investigation of the influence of light intensity could be performed. 

In order to simulate a dark environment, a homemade opaque carton with a removable roof cover and two side holes was necessary, in which all testing procedures were carried out. In a dimly lit test room (30% RH, RT), two quartz bottles were prepositioned in the opaque carton. One quartz bottle was plugged with a rubber stopper and embedded in the gas piping mentioned above, and the other quartz bottle was unoccupied, serving as the air bottle for sensor recovery. When the LED lamp was absent or the power was off, the gas-sensing properties of sensors toward NO_2_ or others in the dark could easily be determined. When the LED lamp was powered on throughout the process, the effect of UV irradiation, including its light intensity, on the sensing performance could likewise be evaluated. It is also worth mentioning that the heating treatment of sensors was put aside due to the real emphasis on UV irradiation, not temperature, in this study. 

In the case of n-type sensing materials (Au/SnO_2_ NWs, for example) and oxidizing target gases (NO_2_, for example), the sensor response is defined as S = R_g_/R_a_ (R_a_ and R_g_ are the dynamically stabilized resistances of the sensor in the air and NO_2_, respectively) in this study. In addition, the time when resistance change reaches 90% during the response and recovery processes is defined as response time (τ_res_) and recovery time (τ_rec_). 

## 3. Results and Discussion

### 3.1. Structural and Morphological Characteristics

**Figure 1:**
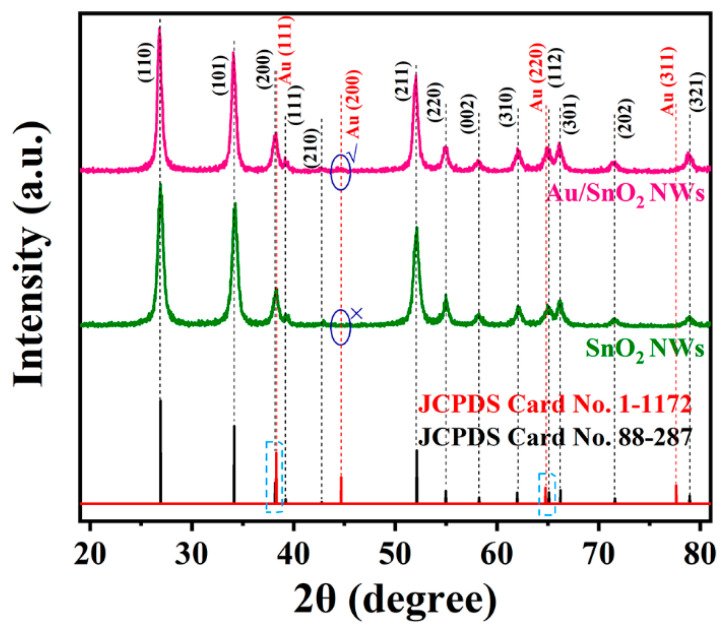
XRD patterns of as-synthesized SnO_2_ and Au/SnO_2_ NWs.

The XRD test is the most direct method for determining the composition information and crystallinity when evaluating a material. As shown in Figure 1, the measured XRD diffraction peaks of SnO_2_ and Au/SnO_2_ NWs are in accordance with tetragonal-phase tin oxide with a lattice constant of a = 4.74 Å, c = 3.19 Å (standard JCPDS card no. 88-287). Although only a weak bump around 44.66° is recognized in the XRD curve of Au/SnO_2_ NWs, it can also be viewed as a strong evidence of the successful Au loading due to its low amount. Through indexing, the Au peak mentioned above coincides with the (200) lattice plane of cubic-phase gold metal with a lattice constant of a = 4.07 Å (standard JCPDS Card No. 1-1172). The disappearance of other Au peaks, such as (111), (220), or (311) is attributed to the overlap with adjacent SnO_2_ peaks or the low intensity. All diffraction peaks of SnO_2_ and Au/SnO_2_ NWs appear sharp and intense, proving their high crystallinity. Visibly, the addition of Au did not hinder the crystal growth of SnO_2_. The corresponding peak positions of Au/SnO_2_ did not shift to the left or right compared with those of SnO_2_, implying their identical lattice parameters. This phenomenon indicates that Au atoms did not enter unit cells of SnO_2_ [21,22,23] due to the ultrahigh chemical stability. 

**Figure 2:**
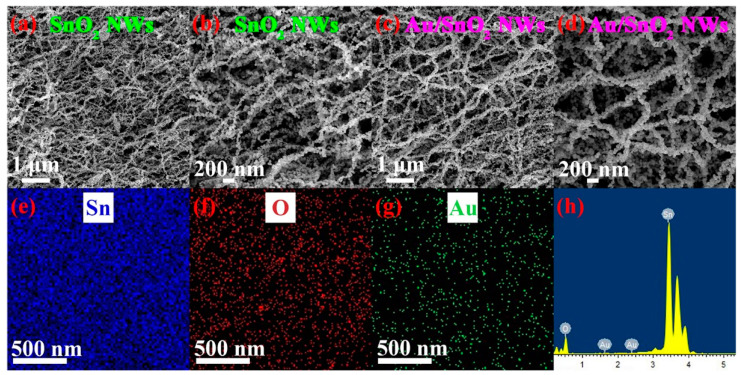
Low- and high-magnification SEM images of (**a**,**b**) SnO_2_ NWs and (**c**,**d**) Au/SnO_2_ NWs. (**e**–**g**) EDS elemental mapping images of Au/SnO_2_ NWs in a certain region of (**c**). (**h**) EDS spectrum of Au/SnO_2_ NWs in a certain region of (**d**).

The internal structure of a substance determines its typical chemical and physical properties. First and foremost, the holistic microstructures of SnO_2_ and Au/SnO_2_ NWs were explored through SEM; the results are presented in Figure 2a–d. As an obvious result, the morphology of SnO_2_ NWs exhibits no significant change before and after the introduction of Au. In low-magnification Figure 2a,c, large quantities of nanowires are intertwined intricately, constructing a three-dimensional structure and leaving abundant interspaces, which is beneficial to the rapid diffusion of gas molecules and sufficient utilization of sensing materials. In high-resolution Figure 2b,d, single nanowires of both SnO_2_ and Au/SnO_2_ exhibit bead-like shapes and rough surfaces, which can provide extra adsorption sites for target gases. However, attached Au particles cannot be observed even in the high-resolution Figure 2d, probably due to the rough surface of Au/SnO_2_ NWs and the tiny size of Au particles. As shown in Figure 2e,f, the EDS elemental mapping analysis on a selected area of Figure 2c shows the uniform and consecutive spatial distribution of Sn and O elements. Particularly, the results in Figure 2g,h verify the presence of Au in the composite. Differently, on account of the low Au concentration, its spatial distribution displayed in Figure 2g is isolated and discrete. 

**Figure 3:**
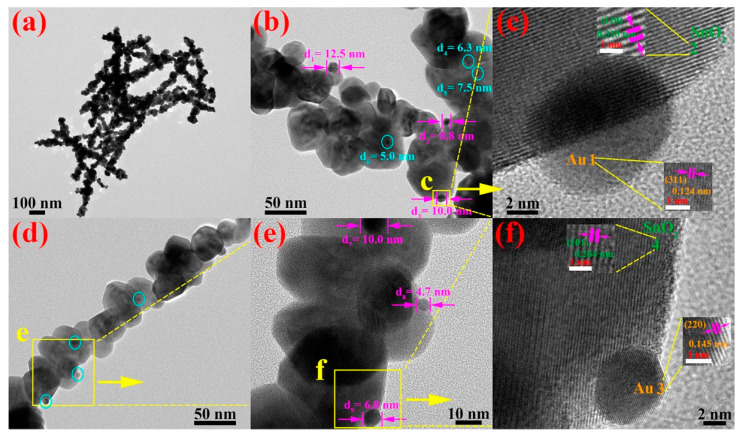
(**a**) Full-view TEM image of Au/SnO_2_ NWs. (**b**,**d**) Locally amplified TEM images of two individual Au/SnO_2_ nanowires. (**c**) HRTEM image of the selected region delimited by a yellow rectangle in (**b**). (**e**) A higher-magnification TEM image of the selected region within a yellow rectangle in (**d**). (**f**) HRTEM image of selected region confined within a yellow rectangle in (**e**).

A more detailed microstructure of Au/SnO_2_ NWs was recorded through TEM and HRTEM techniques. The panoramic Figure 3a contains a dozen of cross-distributed nanowires, possessing an identical bead-like morphology as in Figure 2d. One point worth mentioning is that the lengths of observed nanowires in Figure 3a are 1 μm or less, greatly shortened compared with those in Figure 2c, which are dozens of microns. Broken nanowires in Figure 3a were attributed to weak local binding forces between certain SnO_2_ particles. In the amplified Figure 3b,d, spherical Au particles are depicted. Under the same magnification, the dimensions of partially attached Au particles in Figure 3d are significantly smaller than those in Figure 3b. For the convenience of the measurement, the chosen region delineated by a light-yellow rectangle in Figure 3d was featured in Figure 3e. Thus, nine discernible Au spheres are marked, and their specific diameters are provided in Figure 3b,e. Explicitly, the dimensions of adherent Au spheres differ greatly from each other and it is hard to provide a representative average. Nevertheless, it is certain that the diameters of the vast majority of Au spheres are less than 10 nm, which is small enough for the full release of Au catalytic properties. Analogously, two regions labeled with “c” and “f” in Figure 3b,e were further characterized through HRTEM, giving the lattice information of SnO_2_ and Au, respectively. As shown in Figure 3c,f, Au particles are tightly attached to the surface of SnO_2_ and the boundaries of crystal faces for SnO_2_ and Au are distinct and cognizable. Specifically speaking, calculated d-spacings (orange color) of 0.124 and 0.145 nm correspond to (311) and (220) lattice planes of metallic Au. Likewise, fringe spacings (green color) of 0.335 and 0.264 nm can be attributed to (110) and (101) planes of tetragonal SnO_2_, respectively. 

**Figure 4:**
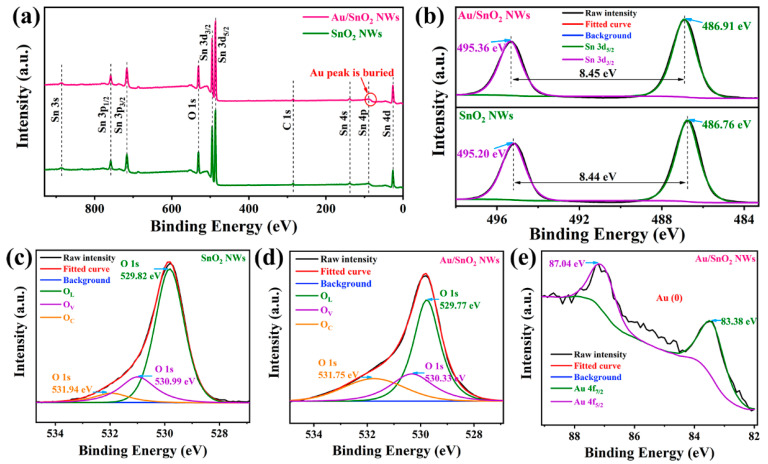
(**a**) Full-scan XPS spectra of SnO_2_ and Au/SnO_2_ NWs. (**b**) High-resolution Sn 3d spectra of SnO_2_ and Au/SnO_2_ NWs. Deconvoluted O 1s core-level spectra of (**c**) SnO_2_ and (**d**) Au/SnO_2_ NWs. (**e**) High-resolution Au 4f spectrum of Au/SnO_2_ NWs.

The XPS test is generally utilized to perform analyses on solid surfaces, involving elemental composition and contents, valence states, chemical bonds, etc. The full XPS spectra of SnO_2_ and Au/SnO_2_ are contrastively displayed in Figure 4a, where element orbital peaks with disparate binding energies are in one-to-one correspondence. Notably, the intrinsic Au 4f peak (85.0 eV) in the Au/SnO_2_ spectrum is buried in the broader Sn 4p peak (90.6 eV) due to the overlap of their binding energies as well as the low Au concentration. The core-level spectra of individual characteristic peaks, Sn 3d, O 1s, and Au 4f, were further analyzed to acquire more information on material composition. For example, in Figure 4b, the Sn 3d spectrum in Au/SnO_2_ is split into independent Sn 3d_5/2_ and 3d_3/2_ peaks, centered at 486.91 and 495.36 eV, respectively [70]. Correspondingly, Sn 3d_5/2_ and 3d_3/2_ peaks in SnO_2_, with identical peak shapes, shifted about 0.15 eV toward lower binding energies, implying internal interactions between Au and SnO_2_. Notably, the differentials of binding energies for the above split peaks in the two materials are kept consistent, about 8.45 eV, which is an eigenvalue for SnO_2_ [71,72].

The content distribution among different oxygen species is of critical importance in the gas-sensing performance of a material. Due to inherent structural characteristics among materials and the consequent differentiation of abilities on the absorption and dissociation of oxygen, contents of active vacancy and chemisorbed oxygen species consistently show a concomitant variation trend. As shown in Figure 4c,d, O 1s core-level spectra of both SnO_2_ and Au/SnO_2_ can be deconvoluted into lattice oxygen (O_L_), oxygen vacancy (O_V_), and chemisorbed oxygen (O_C_) from low to high binding energies. For O_L_ species, their sufficient stabilities as constructional units result in nonparticipation in regular chemical reactions. In contrast, as functional species, O_V_, serving as electron donors, and O_C_, reacting with gas molecules, play a crucial role in the gas-sensing performance of one material. 

On these grounds, some specific indicators concerning oxygen species are summarized in Table 1. As shown, corresponding peaks of the same oxygen species shifted toward lower binding energies in Au/SnO_2_, revealing the impact of Au on the level structure of SnO_2_. Moreover, proportions of O_V_ and O_C_, especially O_C_ species, increased dramatically after the addition of Au, which forebodes the high potential of Au/SnO_2_ NWs when used as gas-sensitive material. Finally, in Figure 4e, binding energies of 83.38 and 87.04 eV, corresponding to Au 4f_7/2_ and 4f_5/2_ peaks, respectively, match well with those reported for metallic Au [21,22,23], thus proving the excellent antioxidative capacity of Au under routine treatment.

### 3.2. Gas-Sensing Properties

**Figure 5:**
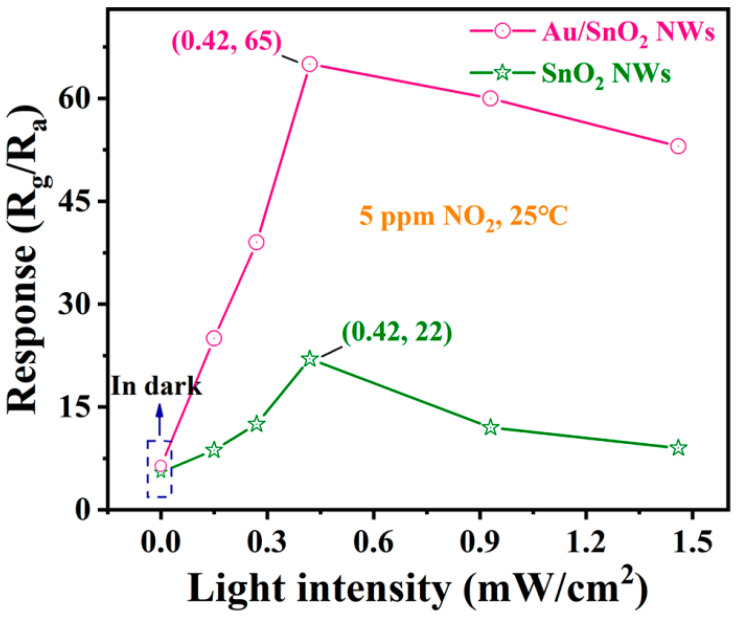
Responses of sensors based on SnO_2_ and Au/SnO_2_ NWs toward 5 ppm NO_2_ at RT in the dark or under UV irradiation with incremental light intensity.

Responses of sensors based on SnO_2_ and Au/SnO_2_ NWs toward 5 ppm NO_2_ at RT with or without UV irradiation were first summarized. As shown in Figure 5, both SnO_2_ and Au/SnO_2_ show a low response toward NO_2_ in the dark. In the blue rectangle, the ordinate of the pink circle (Au/SnO_2_) is slightly over that of the green pentacle (SnO_2_). Visibly, mere embellishment of Au on SnO_2_ did not lead to a qualitative improvement in the sensing performance toward NO_2_ in the dark at RT. In contrast, the introduction of UV irradiation instantaneously triggered the differentiation of two curves, the tendency becoming more distinct with the increase in light intensity. To be sure, UV irradiation is much more effective in enhancing the sensor response toward NO_2_ than Au modification. At the same time, the range of response elevation for Au/SnO_2_ by UV irradiation with the same intensity is significantly greater than that for SnO_2_. In other words, the existence of Au is an important premise for fully exploiting the function of UV irradiation, and internal interactions exist between Au and UV irradiation, which further improved the sensor response. Specifically speaking, the optimal light intensity of UV irradiation for both SnO_2_ and Au/SnO_2_ is the same, i.e., 0.42 mW/cm^2^, within the existing test accuracy. To the left of the highest point, the pink curve (Au/SnO_2_) is much steeper and its maximum response equals 1015% of that in the dark. Correspondingly, the amplification for the green curve (SnO_2_) is only 400%. To the right of the highest point, the downward trend for the pink line (Au/SnO_2_) is much slower. After calculation, the responses of Au/SnO_2_ and SnO_2_ NWs under UV irradiation of 1.46 mW/cm^2^ decreased by 18.5 and 59.1%, respectively, compared with their maximums at 0.42 mW/cm^2^.

**Figure 6:**
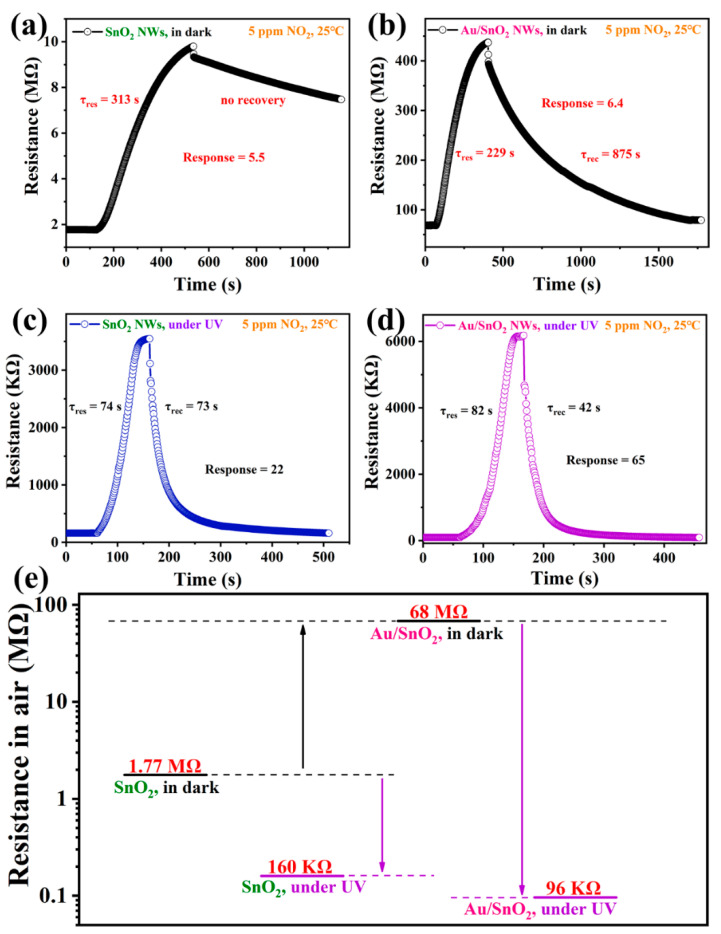
Single-cycle response–recovery transient curves of the sensor based on SnO_2_ NWs toward 5 ppm NO_2_ at RT (**a**) in the dark and (**c**) under UV irradiation (0.42 mW/cm^2^). Single-cycle response–recovery transient curves of the sensor based on Au/SnO_2_ NWs toward 5 ppm NO_2_ at RT (**b**) in the dark and (**d**) under UV irradiation (0.42 mW/cm^2^). (**e**) The dynamically stable resistance values in the air of sensors based on SnO_2_ and Au/SnO_2_ NWs in the dark and under UV irradiation (0.42 mW/cm^2^).

As shown in Figure 6a–d, at RT, singly periodic response–recovery curves of sensors based on SnO_2_ and Au/SnO_2_ NWs toward NO_2_ in the dark and under UV irradiation with optimal intensity (0.42 mW/cm^2^) were provided in sequence. Excluding responses already presented in Figure 5, more emphasis will be placed on the analysis of the response/recovery time of sensors under different conditions, shown in Figure 6a–d. In Figure 6a, the response/recovery properties of the sensor based on SnO_2_ NWs are terrible in the dark at RT, demonstrating mediocre responses. This sensor presents a lengthy response time of 313 s. In particular, up to 700 s, the sensor is barely able to recover 28.8% of the total resistance change. It is observed that the sensor cannot spontaneously complete its recovery process in the dark without external aids, such as optical or thermal excitation, which is common in NO_2_ sensing due to its strong chemical affinity. However, in the dark and at RT as well, the response time of the sensor based on Au/SnO_2_ NWs is shortened to 229 s. More importantly, the Au/SnO_2_ sensor can achieve complete recovery in 1370 s, which, although still long, is a great improvement compared with that of the SnO_2_ sensor. Compared with the limited improvements in sensor responses in the dark at RT, the enhancing effect of Au through its catalytic ability on the response/recovery properties of the sensor is more significant. 

As shown in Figure 6a–d, when keeping the sensitive material consistent, UV irradiation can promote the response and response/recovery properties by a wide margin at the same time. In Figure 6c,d, both sensors display much shorter response times and concurrently implement complete and quick recovery. Comparing the data in Figure 6b,c with those in Figure 6a, the effect of UV irradiation on the improvement of response/recovery properties is significantly superior to Au loading. Furthermore, when comparing the results in Figure 6c,d, a combination of Au loading and UV irradiation can not only continue to enhance the sensor response but also shorten the recovery time to a great extent, which is hard-won considering that the recovery time of 73 s in Figure 6c is already short. It is worth mentioning that the response time in Figure 6d is slightly longer than that in Figure 6c. To some extent, this is reasonable in view of the much larger range of resistance changes in Figure 6d, which will consume considerably more time under the same conditions [44]. 

We preliminarily explored the changes in the initial resistance of the sensors at RT in the air in the four cases shown in Figure 6a–d. Firstly, in the dark, the initial resistance of SnO_2_ NWs at RT in the air is 1.77 MΩ. After Au loading, the metal–semiconductor contact generated a Schottky barrier, which increased the initial resistance of Au/SnO_2_ to a much higher value of 68 MΩ. As expected, the initial resistance values of both sensors dropped precipitously due to a surge of charge carriers excited by UV irradiation. In addition, the coexistence of Au loading and UV irradiation yielded a much greater degree of resistance drop, which is apparently caused by the LSPR effect between Au particles and UV irradiation. More specifically, the resistance for Au/SnO_2_ and SnO_2_ NWs decreased 11-fold (1.77 MΩ→160 KΩ) and 708-fold (68 MΩ→96 KΩ), respectively. 

**Figure 7:**
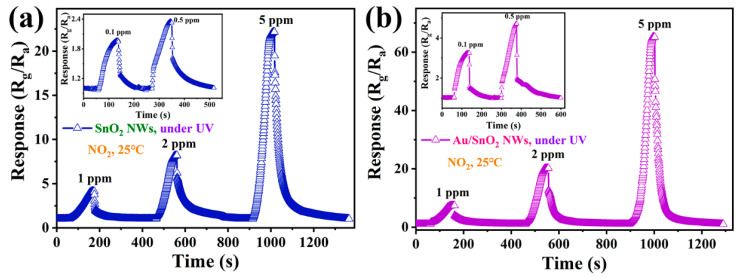
Dynamic response–recovery curves of sensors based on (**a**) SnO_2_ and (**b**) Au/SnO_2_ NWs to NO_2_ with concentrations in the range of 0.1–5 ppm at RT under UV irradiation (0.42 mW/cm^2^).

Due to the proven outstanding performance of two sensors toward NO_2_ under UV irradiation, the response–concentration properties continued to be evaluated under the above conditions. As shown in Figure 7a,b, overall, UV irradiation can assist the implementation of holonomic response processes over a wide NO_2_ concentration range, and the responses of the two sensors maintain rapid growth with the increases in NO_2_ concentration. Moreover, the two sensors show good potential in low-concentration NO_2_ sensing; their responses toward 0.1 ppm NO_2_ reached 1.95 and 3.23, respectively. By rough calculation, from 0.1 to 5 ppm, the responses to NO_2_ concentrations for SnO_2_ and Au/SnO_2_ sensors are 1.19-, 1.79-, 1.97-, and 2.68-fold and 1.46-, 1.62-, 2.65-, and 3.21-fold, respectively, higher than those to prior NO_2_ concentrations. Relatively speaking, the above results reflect the better performance of Au/SnO_2_ under UV irradiation compared with SnO_2_.

**Figure 8:**
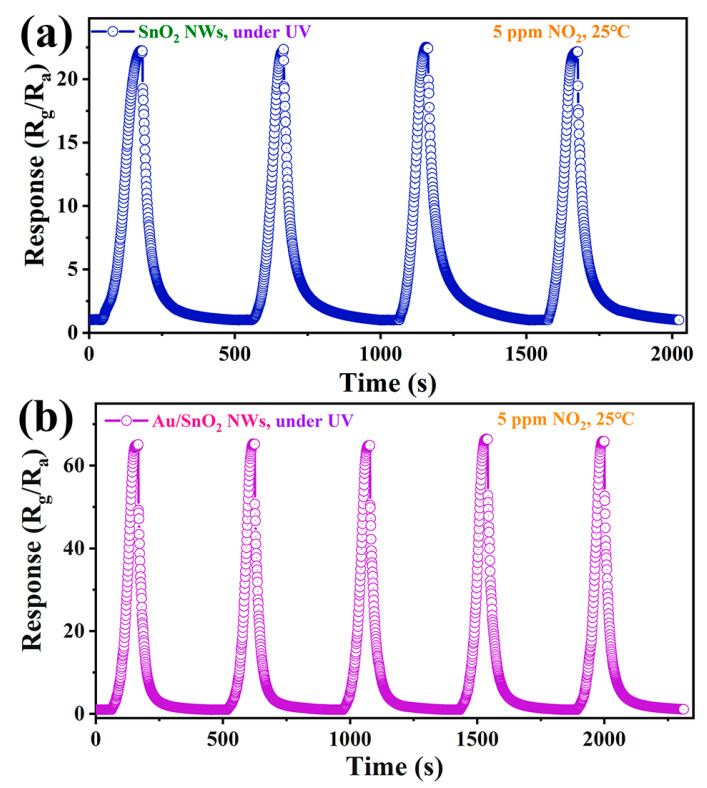
The cyclic response–recovery curves of sensors based on (**a**) SnO_2_ and (**b**) Au/SnO_2_ NWs to 5 ppm NO_2_ at RT under UV irradiation (0.42 mW/cm^2^).

Under UV irradiation, repeatability tests of the two sensors toward 5 ppm NO_2_ at RT were performed. The reproducibility of data is very necessary and acts as strong evidence of result objectivity. As shown in Figure 8a,b, many key points of curves for SnO_2_ or Au/SnO_2_ sensors are analogical, involving the amplitudes and gradients. Visibly, relevant performance indicators can be reproduced absolutely within a certain range of error. These findings verified the availability and reliability of UV irradiation in continuously ensuring the optimization and stable output of sensor performance. 

**Figure 9:**
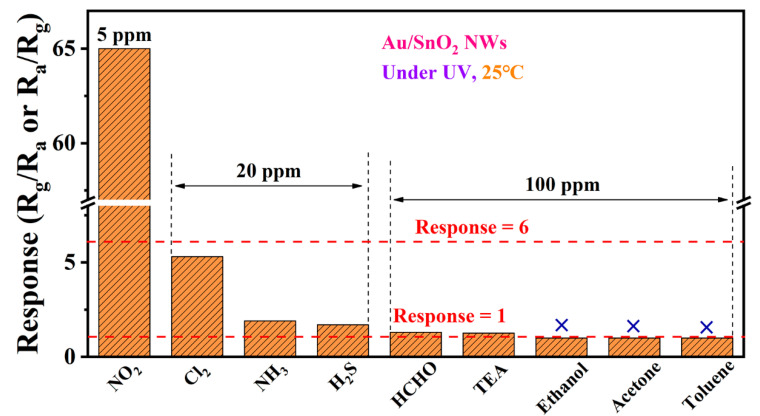
Responses of the sensor based on Au/SnO_2_ NWs toward 5 ppm NO_2_ and common interfering gases with higher concentrations at RT under UV irradiation (0.42 mW/cm^2^).

Taking Au/SnO_2_ NWs as an example, the selectivity of the sensor at RT under UV irradiation was expounded. In Figure 9, test gases were classified into three categories: NO_2_, other gaseous gases (Cl_2_, NH_3_, and H_2_S), and volatile organic compounds (VOCs, from HCHO to toluene). To visualize data, responses of interferential gases were intentionally amplified by increasing their detection concentrations. Nevertheless, responses of the sensor based on Au/SnO_2_ NWs vary considerably among three categories. For example, ratios of the response toward 5 ppm NO_2_ to those toward gases in the second category (20 ppm) range from 12.26 (Cl_2_) to 38.24 (H_2_S). Moreover, although the concentration of VOCs is up to 100 ppm, the sensor still shows weak responses toward HCHO and TEA, and even no detectable responses toward the three residual VOCs. As it turned out, Au/SnO_2_ NWs exhibit excellent selectivity toward NO_2_ at RT under UV irradiation.

**Figure 10:**
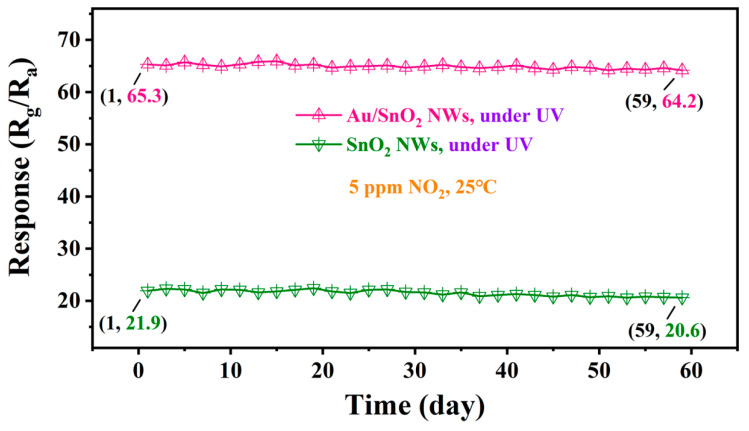
Long-term stability tests of sensors based on SnO_2_ and Au/SnO_2_ NWs to 5 ppm NO_2_ at RT under UV irradiation (0.42 mW/cm^2^).

To examine the performance sustainability of two sensors under UV irradiation, the responses of two sensors toward 5 ppm NO_2_ at RT were regularly recorded every other day for two months. As depicted in Figure 10, responses in the two curves maintain a dynamic fluctuation, slightly declining but stable overall. Two months later, the responses of sensors based on SnO_2_ and Au/SnO_2_ NWs remained at 94.06% and 98.32% of their initial values, respectively, strongly demonstrating the good long-term stability of device performance under UV irradiation.

In addition, a comprehensive comparison between the sensing performance of the Au/SnO_2_ NWs sensor fabricated in this study and other recent reports on room-temperature NO_2_ gas sensors [32,33,34,40,41,53,62,64,73,74,75] is presented in Table 2. In contrast, the performance indexes exhibited by Au/SnO_2_ NWs sensor were considered distinguished on the whole.

### 3.3. Gas-Sensing Mechanism

**Figure 11:**
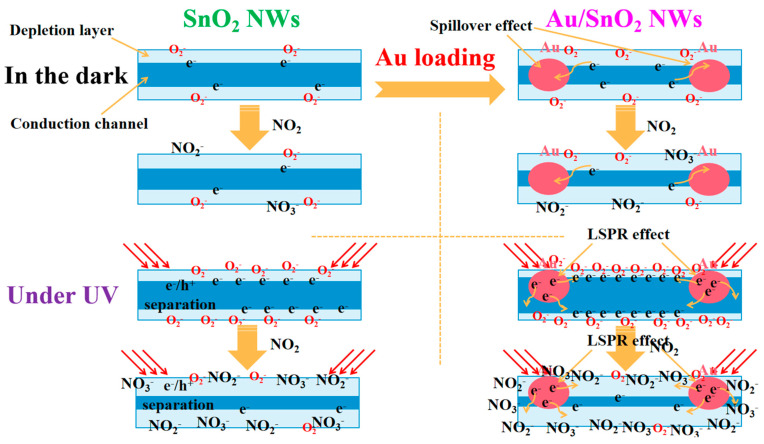
Schematic illustration of the sensing mechanism in this study.

The internal mechanisms of resistance variation shown in Figure 6e must be thoroughly understood, which is in direct correlation with the sensing performance in different cases. In order to make the related analysis more intuitive, a schematic diagram of the mechanism has been given in Figure 11.

After Au loading, the initial resistance increased from 1.77 MΩ for pure SnO_2_ to 68 MΩ for Au/SnO_2_ at RT in the dark. In metal–semiconductor contact, a Schottky barrier will form at interfaces of Au and SnO_2_ due to the difference in their work functions [64]. Specifically, the work functions of Au and SnO_2_ were reported to be 5.1 [43,64] and 4.5~4.75 eV [33,46,48,53,54], respectively. Then, electrons will spontaneously flow from SnO_2_, with a lower work function, to Au, with a higher work function [33,43,46,64], resulting in the generation of a depletion layer and an increase in resistance. When detecting oxidizing NO_2_ through n-type materials, a higher R_a_ is not considered disadvantageous despite the response equation being R_g_/R_a_ and considering that a lower carrier concentration tends to produce a greater proportion of variation under equal conditions.

In general, chemical sensitization is regarded to play a vital role in improving sensing properties for Au-catalyzed systems. On the one hand, Au nanoparticles can facilitate the chemisorption and dissociation of both target gas and oxygen due to their high catalytic efficiencies [21,22,43]; on the other hand, all dissociated species will be rapidly transferred from the Au nanoparticles to the oxide surface due to the well-known spillover effect of Au [21,22,23,43]. In fact, the increase in O_C_ content due to Au catalyzation has been proven in Table 1. As shown, Au, acting as a medium, can enhance the quantity and efficiency of reactions occurring at the surface of metal oxide, which reasonably explains the improved sensing performance in Figure 6b compared with that in Figure 6a. However, the increased amplitude of sensing performance in this study is very limited, which indicates the suppression of the effects of Au at low temperatures. 

Under UV irradiation (0.42 mW/cm^2^), the resistance of pure SnO_2_ is sharply reduced from 1.77 MΩ to 160 KΩ, which can be attributed to the role of photoexcitation. When the photon energy of the excitation source exceeds bandgaps of SMOs, the occurrence of electron transition will lead to the generation of electron–hole pairs [38,44]. First, photoinduced holes will react with O_C_ on the surface, resulting in the desorption of O_C_ in the form of O_2_ [43,44], which will decrease sensor resistance due to the backflow of electrons [43]. In fact, a portion of abundant photogenerated electrons will transform more oxygen into O_C_, which is far greater than the O_C_ loss [42,43]. As a result, the content of O_C_ in pure SnO_2_ will increase greatly under UV irradiation, leading to a clear increase in NO_2_ reactions and then a much higher sensor response. At the same time, the surplus hot electrons derived from UV excitation will react directly with NO_2_. In addition, the high activity and mobility of photoinduced electrons contribute considerably to sensor response/recovery properties. These theories can interpret changes in all important indicators between Figure 6a,c.

Finally, the decreased amplitude of resistance from 68 MΩ in the dark to 96 KΩ under UV irradiation for Au/SnO_2_ is much higher than that for pure SnO_2_. Distinctly, the LSPR effect of Au under UV irradiation plays a decisive role in this process [43,62,63]. The light-excited resonant electrons are sufficiently active to overcome the Schottky barrier, escaping from Au nanoparticles to the conduction of SnO_2_ [43,56,62,63,64]. Compared with the case in Figure 6c, the free resonant electrons in Au/SnO_2_ under UV irradiation are much more abundant, with much faster mobility and much higher activity, accounting for the better sensing performance in Figure 6d [43,62].

## 4. Conclusions

In conclusion, Au/SnO_2_ NWs were successfully prepared through the in situ modification of Au nanoparticles on SnO_2_ NWs in the process of electrospinning. Some essential characterizations were conducted to verify its structure and feature. The RT gas-sensing properties toward NO_2_ of pure SnO_2_ and Au/SnO_2_ were rigorously explored and analyzed in the dark and under UV irradiation. Based on relevant experimental data, the gas-sensing mechanism was reasonably proposed, clearly detailing the specific enhancement theory in every case. Among all cases, the optimal sensing properties toward NO_2_ for Au/SnO_2_ under UV irradiation were attributed to the LSPR effect of Au. This study is of great significance to RT photoexcited NO_2_ sensing.

## Figures and Tables

**Table 1:** The conclusive fitting results of O 1s XPS spectra of SnO_2_ and Au/SnO_2_ NWs.

Materials	Oxygen Species	Binding Energy (eV)	Relative Percentage (%)	O_V_ + O_C_ (%)
SnO_2_ NWs	O_L_ (Sn–O)	529.82	74.22	25.78
	O_V_ (vacancy)	530.99	19.09	
	O_C_ (chemisorbed)	531.94	6.69	
Au/SnO_2_ NWs	O_L_ (Sn–O)	529.77	52.81	47.19
	O_V_ (vacancy)	530.33	22.51	
	O_C_ (chemisorbed)	531.75	24.68	

**Table 2:** A comparison of RT NO_2_ sensing performance from the sensor developed in this study and those in other studies.

Materials	Temp. (°C)	Light Source (nm)	Conc. (ppm)	Response	τ_res._/τ_recov._ (s)	Year	Ref.
SnS_2_	RT	Vis. (520–550)	8	10.8 ^a^	164/236	2020	[32]
SnO_2/_SnS_2_	RT	Vis. (450)	0.2	5.3 ^a^	950/1160	2020	[33]
SnS_2_	RT	Vis. (450–455)	5	14.28 ^b^	400/1100	2021	[34]
Au/ZnO	RT	Vis. (532)	1	4.66 ^a^	~400/~300 ^f^	2020	[62]
ZnO/g-C_3_N_4_	RT	Vis. (460)	7	44.8 ^a^	142/190	2020	[73]
Graphene	RT	UV (370)	1	25% ^c^	~600/~200 ^f^	2019	[40]
ZnO/TiO_2_	RT	UV (365)	5	105% ^d^	26/224	2020	[41]
rGO/SnO_2_	RT	UV (365)	0.5	23% ^e^	426/438	2019	[53]
Au/ZnO	RT	UV (365)	1	2.3 ^a^	160/370	2020	[64]
SnSe_2_/SnO_2_	RT	UV (–)	10	9.53 ^a^	80/144	2022	[74]
ZnO/MoS_2_	RT	UV (365)	10	293% ^e^	258/72	2020	[75]
Au/SnO_2_	RT	UV (365–370)	5	65 ^a^	82/42	-	This study
			1	7.4 ^a^	73/65		
			0.1	3.25 ^a^	43/41		

Temp.: Operating temperature; Conc.: Gas concentration; τ_res._/τ_recov._: response/recovery time; Ref.: references; Vis.: visible light. ^a^: R_g_/R_a_; ^b^: (R_g_ − R_a_)/R_a_; ^c^: (R_a_ − R_g_)/R_a_ × 100%; ^d^: R_g_/R_a_ × 100%; ^e^: (R_g_ − R_a_)/R_a_ × 100%; ^f^: inferred value, not measured value.

## Data Availability

The data that support the findings of this study are available from the first author or corresponding authors upon reasonable request.
